# Combined ACL and ALL Reconstruction Using Allografts as the ACL Graft Source Reduces Surgical Failure and Improves Graft Maturity Compared with Isolated ACL Reconstruction

**DOI:** 10.3390/jcm15020735

**Published:** 2026-01-16

**Authors:** Hyun-Soo Moon, Sungjun Kim, Min Jung, Kwangho Chung, Se-Han Jung, Junhee Cho, Gyunghyun Shin, Sung-Hwan Kim

**Affiliations:** 1 Arthroscopy and Joint Research Institute, Yonsei University College of Medicine, Seoul 03722, Republic of Korea; 2 Department of Orthopedic Surgery, Gangnam Severance Hospital, Yonsei University College of Medicine, Seoul 06273, Republic of Korea; 3 Department of Radiology, Gangnam Severance Hospital, Yonsei University College of Medicine, Seoul 06273, Republic of Korea; 4 Department of Orthopedic Surgery, Severance Hospital, Yonsei University College of Medicine, Seoul 03722, Republic of Korea; 5 Department of Orthopedic Surgery, Yongin Severance Hospital, Yonsei University College of Medicine, Yongin 16995, Republic of Korea

**Keywords:** anterior cruciate ligament reconstruction, anterolateral ligament reconstruction, allograft, surgical failure, graft maturity

## Abstract

**Objectives:** This study aimed to perform matched comparisons of the surgical outcomes of combined anterior cruciate ligament (ACL) and anterolateral ligament (ALL) reconstruction with those of isolated ACL reconstruction, in which allografts were used for the ACL. **Methods:** Patients who underwent anatomical ACL reconstruction with or without additional ALL reconstruction between 2017 and 2023 and had a minimum follow-up of 2 years were included and grouped according to whether an additional ALL reconstruction was performed. The cohorts were statistically adjusted using an inverse probability of treatment weighting (IPTW) to control for potential confounders related to surgical indication, including age, activity level, sex, rotational knee laxity, and preoperative osteoarthritic grade. Between-group comparisons were conducted for baseline characteristics, clinical outcomes, knee laxity, and radiologic parameters. **Results:** Fifty-nine patients were included (Group 1: 39 isolated ACL reconstructions; Group 2: 20 combined ACL and ALL reconstructions). Before IPTW adjustment, a significant difference was observed in the preoperative pivot-shift test (*p* = 0.008), which was no longer significant after weighting. Postoperative functional outcomes and knee stability were comparable between groups; however, the incidence of surgical failure was significantly lower in Group 2 both before and after IPTW adjustment (*p* = 0.044 and *p* = 0.049, respectively). Regarding radiologic parameters, the signal-to-noise quotient of the ACL graft was also significantly lower in Group 2, both before and after IPTW adjustment (*p* = 0.046 and *p* = 0.038, respectively). **Conclusions:** In ACL reconstruction using allografts, the addition of ALL reconstruction resulted in more favorable clinical and radiologic outcomes—particularly a lower incidence of surgical failure and greater postoperative graft maturity—compared with isolated ACL reconstruction.

## 1. Introduction

The addition of anterolateral augmentation procedures, including anterolateral ligament (ALL) reconstruction and lateral extra-articular tenodesis (LET), to anterior cruciate ligament (ACL) reconstruction has been reported to provide significant clinical benefits [1,2,3,4,5]. Combined procedures have demonstrated superior outcomes compared with isolated ACL reconstruction, not only in terms of functional recovery and residual knee laxity but also with respect to graft failure and reoperation rates [1,2,3,4,5,6]. Consequently, the use of anterolateral augmentation procedures in conjunction with ACL reconstruction has been steadily increasing, with both ALL reconstruction and LET being applied according to surgeon preference and patient characteristics [7,8,9].

However, most previous studies on combined ACL and anterolateral augmentation procedures have been limited to cases in which autografts were used as the graft source for ACL reconstruction [1,2,3,4,5], and the clinical outcomes of allograft-based procedures remain largely uninvestigated. Allografts have been reported to require a relatively longer period for ligamentization than autografts and may yield less favorable outcomes, particularly in young and active individuals [10,11,12,13,14,15]. In this regard, graft maturation may be influenced not only by mechanical stability but also by biological healing processes originating from the surrounding soft tissues [16]. Given that the biological behavior of allografts differs from that of autografts, the benefits of anterolateral augmentation observed in autograft-based studies cannot be assumed to translate directly to allograft ACL reconstruction. With the widespread use of allografts in ACL reconstruction worldwide [13,17,18], there remains a need to clarify the clinical outcomes of combined ACL and anterolateral augmentation procedures when ACL reconstruction is performed using allografts. Furthermore, a direct comparison with isolated ACL reconstruction is warranted to better delineate the potential advantages of this combined approach.

Therefore, the purpose of this study was to perform matched comparisons of the surgical outcomes of combined ACL and ALL reconstruction with those of isolated ACL reconstruction, in which allografts were used for the ACL. It was hypothesized that combined ACL and ALL reconstruction would yield superior subjective and objective outcomes compared with isolated ACL reconstruction.

## 2. Methods

**Figure 1:**
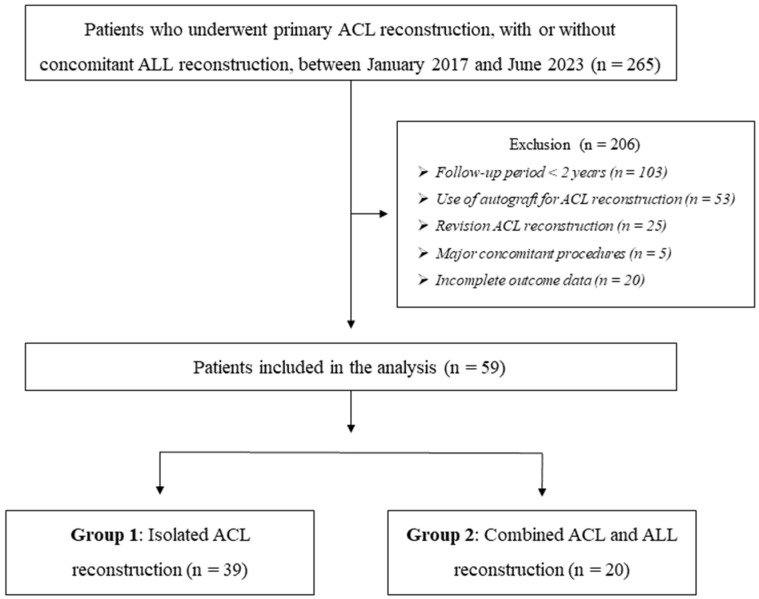
Flowchart of patient selection and grouping.

Approval for this study was obtained from the Institutional Review Board of Gangnam Severance Hospital, and the requirement for informed consent was waived owing to the retrospective design of the study (IRB No. 3-2025-0279). A retrospective review of electronic medical records was conducted for patients who underwent surgical treatment for ACL rupture at a single institution by a single senior surgeon between January 2017 and June 2023, which served as the study timeframe for case inclusion. Patients who underwent anatomical ACL reconstruction using the transportal technique were eligible for inclusion, regardless of whether the procedure was performed as an isolated ACL reconstruction or in combination with ALL reconstruction, provided that an allograft was used as the graft source for the ACL. Patients were excluded if they met any of the following criteria: (1) the follow-up period was less than two years; (2) an autograft was used for ACL reconstruction; (3) surgery was performed as a revision procedure; (4) major concomitant procedures such as meniscal allograft transplantation; multi-ligament reconstruction other than ALL reconstruction, or osteotomy; or (5) the available outcome data were insufficient. After applying these criteria, the eligible patients were divided into two groups according to whether ALL reconstruction was performed in addition to ACL reconstruction: Group 1 comprised patients who underwent isolated ACL reconstruction, and Group 2 comprised those who underwent combined ACL and ALL reconstruction (Figure 1).

### 2.1. Surgical Procedures and Rehabilitation Methods

Before surgery, all patients received detailed counseling regarding the available graft options for ACL reconstruction—including autogenous bone–patellar tendon–bone grafts, autogenous hamstring tendon grafts, and allogeneic tendon grafts—and were informed of the potential advantages and disadvantages of each option [19,20]. The final choice of graft type was made by the patient. For allograft reconstruction, a low-dose γ-irradiated (12.8–12.9 kGy, cobalt-60) tibialis anterior allograft measuring 8–10 mm in diameter was utilized. The graft was thawed at room temperature in a saline solution containing antibiotics, after which both ends were whipstitched with No. 1 Ethibond sutures (Ethicon, Somerville, NJ, USA). The diameter of the tendon was determined by folding the graft over an Ethibond suture to create a double-strand configuration and passing it through a calibrated sizing block (GraftMaster III; Smith & Nephew, Watford, UK). The prepared graft was then pre-tensioned to approximately 80 N for 20 min on a graft-preparation station (GraftMaster III; Smith & Nephew, Watford, UK) [21].

ACL reconstruction began with diagnostic arthroscopy performed through a parapatellar high anterolateral portal to evaluate all knee compartments [22]. Meniscal and/or chondral lesions, if present, were treated appropriately before ligament reconstruction. A tibial tunnel was then created at the anatomic tibial footprint of the ACL, with a diameter matching that of the prepared allograft. An independent femoral tunnel was drilled via a far anteromedial portal at approximately 120° of knee flexion to ensure adequate length and prevent posterior wall blowout [23]. The femoral socket was prepared to match the graft size and extended proximally to accommodate the EndoButton CL (Smith & Nephew, Watford, UK). The graft was inserted and fixed to the femur using the EndoButton, and proper flipping was confirmed. Distal fixation was achieved after pre-tensioning the graft to 80 N and cycling it 20 times. The graft was then secured with a bioabsorbable interference screw and an additional cortical screw with a washer (ConMed Linvatec, Largo, FL, USA) at approximately 10–20° of knee flexion.

**Figure 2:**
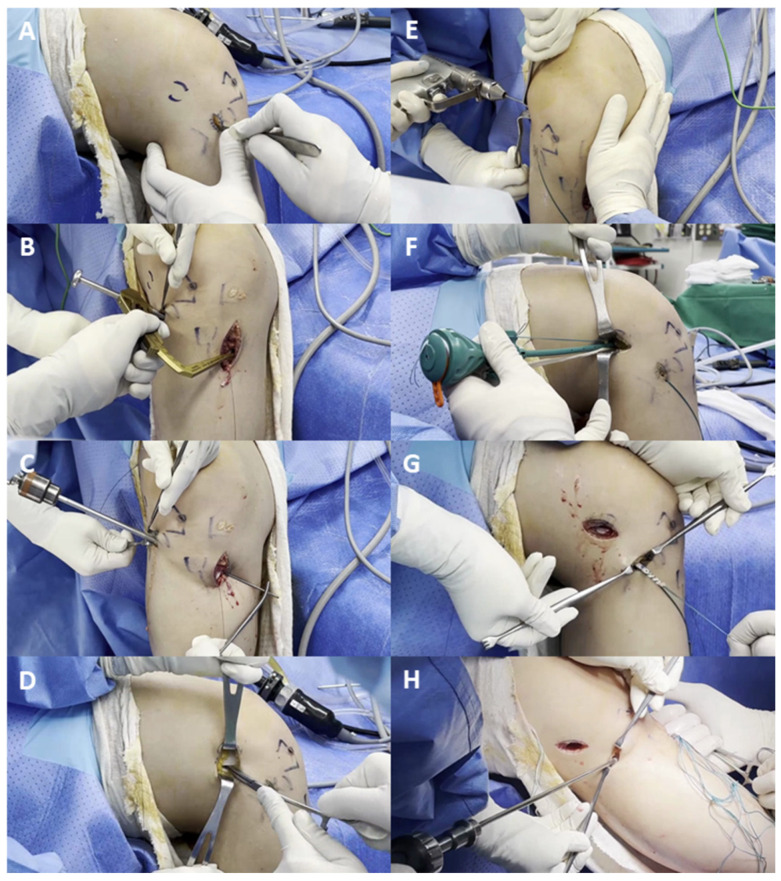
Surgical procedure of anterolateral ligament reconstruction using the single-arm technique: (**A**) A short vertical incision was made midway between Gerdy’s tubercle and the anterior margin of the fibular head. (**B**,**C**) A 6 mm tibial tunnel was reamed toward the opposite cortex at an orientation of 10° axial and −30° coronal to minimize the risk of saphenous nerve injury. (**D**) On the femoral side, a small incision was made over the lateral femoral epicondyle, and the point just posterosuperior to the epicondyle was identified beneath the iliotibial band. (**E**) Thereafter, a 5–6 mm diameter, 30 mm deep tunnel was reamed with an orientation of 0° axial and −40° coronal to avoid convergence with the anterior cruciate ligament femoral tunnel. (**F**) The whipstitched graft was fixed to the femoral tunnel with a 4.5–5.5 mm SwiveLock^®^ anchor. (**G**) The graft was passed deep to the iliotibial band and advanced into the tibial tunnel after distal whipstitching. (**H**) Tibial fixation was completed with a bioabsorbable interference screw under gentle traction (~20 N) in near-full extension.

At our institution, ALL reconstruction began to be performed actively around 2019. The general surgical indications for performing additional ALL reconstruction in conjunction with ACL reconstruction included the following: (1) young patients with a high activity level, (2) grade 2 or 3 pivot shift, and (3) revision ACL reconstruction [24,25,26]. The ALL reconstruction was performed using either a single-arm or an inverted V-shaped double-arm configuration, with most cases employing the single-arm technique. A tibialis anterior allograft was used, trimmed to approximately 5 mm in diameter, and whipstitched for about 30 mm at one end with No. 2 Ethibond sutures. On the tibial side, a short vertical incision was made just posterior to Gerdy’s tubercle. The tibial fixation was aimed midway between Gerdy’s tubercle and the anterior margin of the fibular head [27]. After guide pin insertion, a 6 mm tibial tunnel was reamed toward the opposite cortex, with an orientation of 10° axial and −30° coronal [28]. The femoral fixation was targeted at the anatomic origin of the ALL, located just proximal and posterior to the lateral femoral epicondyle [27,29]. A guide pin was inserted distally in the coronal plane, oriented at 0° axial and −40° coronal, to prevent convergence with the ACL femoral tunnel [30], and a closed-socket tunnel approximately 30 mm in length was created. The whipstitched graft end was then secured to the femoral socket with a 4.5–5.5 mm SwiveLock^®^ anchor (Arthrex, Naples, FL, USA). The graft was then passed deep to the iliotibial band and routed through the extra-articular layer from the femur to the tibia. The distal portion of the graft (approximately 30 mm) was whipstitched with No. 2 Ethibond sutures, and the graft was then advanced into the tibial tunnel along the pre-inserted guide pin. Tibial fixation was achieved using a bioabsorbable interference screw while applying gentle traction (~20 N) with the knee in near full extension (10–20° flexion, neutral rotation) [31] (Figure 2).

Postoperative rehabilitation followed a standardized protocol regardless of whether combined ALL reconstruction was performed. Immediately after surgery, patients were allowed crutch-assisted ambulation with tolerable weight-bearing. Early exercises for passive knee range of motion and isometric quadriceps strengthening were encouraged from the first postoperative day. At 6 weeks postoperatively, full weight-bearing ambulation was permitted, and closed kinetic chain exercises were initiated. In cases where meniscal or chondral repair was performed, both weight-bearing and knee range of motion exercises were delayed in accordance with the corresponding rehabilitation protocol for meniscal or cartilage repair. After 6 months, patients were allowed to begin open kinetic chain exercises, jogging, and swimming. Return to sports activities involving pivoting, jumping, or side-stepping was permitted approximately 9 months after surgery.

### 2.2. Patient Assessment

The evaluation of patients involved conducting clinical assessments, encompassing demographic data, functional scores, and knee laxity, along with intraoperative data and various radiographic parameters. Functional scores used for patient evaluation included the International Knee Documentation Committee (IKDC) subjective, Lysholm, and Tegner activity scores [32,33]. The assessment of knee laxity was conducted for both anterior-posterior (AP) and rotational laxity. AP laxity of the knee was measured using a KT-2000 arthrometer (MEDmetric, San Diego, CA, USA) under maximum manual force [34], as well as with the manual Lachman test. The anterior tibial translation of the affected knee was measured using a KT-2000 arthrometer and compared with that of the contralateral knee; the side-to-side difference (SSD) was recorded for analysis. Rotational laxity of the knee was evaluated through the manual pivot shift test and recorded as follows: grade 0 (absent = normal), grade 1 (glide = nearly normal), grade 2 (jump = abnormal), and grade 3 (transient lock = severely abnormal). The assessments of functional scores and knee laxity were based on records conducted independently of this study when patients visited outpatient clinics. In this study, the analysis was performed using records from the preoperative phase, postoperative 2-years, and the final follow-up. Surgical failure after ACL reconstruction was defined as the presence of any of the following findings indicative of unfavorable surgical outcomes during follow-up: (1) graft re-tear confirmed by magnetic resonance imaging (MRI); (2) AP knee laxity graded as 2 or 3, based on KT-2000 arthrometer measurements or the manual Lachman test; or (3) rotational knee laxity graded as 2 or 3 on the manual pivot-shift test [35,36,37]. Although surgical failure did not necessarily correspond to the need for revision surgery at our institution—since the decision for revision was also influenced by patient characteristics and the severity of subjective symptoms—this definition was adopted to comprehensively capture all potential cases associated with unsatisfactory postoperative outcomes.

Intraoperative data were analyzed with respect to the diameter of the ACL graft and the intra-articular status of the meniscus and articular cartilage, as documented in records completed immediately after surgery. The condition of the meniscus was assessed separately for the medial and lateral compartments and categorized as functional, nonfunctional, or repaired. A meniscus was regarded as functional when no pathologic tear was present or when a limited partial meniscectomy had been performed for lesions not compromising its biomechanical integrity [38]. A nonfunctional meniscus referred to cases with a history of prior subtotal meniscectomy or the presence of extensive, irreparable tears [38]. Tibiofemoral articular cartilage was classified as intact, low-grade lesion, high-grade lesion, or treated with a cartilage repair procedure. Lesions corresponding to International Cartilage Repair Society grades 1–2 were considered low-grade, whereas those graded 3 or higher were defined as high-grade defects.

Radiologic assessment was conducted using both plain radiographs and MRI. On plain radiographs, the degree of osteoarthritis was evaluated on standing AP knee views according to the Kellgren–Lawrence grading system. The posterior tibial slope and lateral femoral condyle ratio were measured on lateral knee views, and the hip–knee–ankle (HKA) angle was determined on full-length weight-bearing lower extremity AP radiographs [39,40,41,42]. Radiographic osteoarthritis grade and HKA angle were assessed at three time points: preoperatively, 2 years postoperatively, and at the final follow-up. MRI evaluation included an assessment of ACL graft maturity. One year after surgery, 3.0-T MRI scans were obtained using either an Achieva system (Philips Healthcare, Best, The Netherlands) or a Discovery 750w system (GE Healthcare, Waukesha, WI, USA). Turbo spin-echo T2-weighted sequences with a slice thickness of 3 mm were used for the analyses. Graft signal intensity (SI) was measured at the proximal, middle, and distal thirds of the graft according to the method described by Tashiro et al. [43]. The signal-to-noise quotient (SNQ) was then calculated to quantify graft maturity using the following formula: SNQ = (SI of ACL graft—SI of posterior cruciate ligament)/SI of the background [43]. Radiologic measurements were independently performed on a picture archiving and communication system (PACS) workstation by two orthopedic surgeons experienced in radiologic assessment of the knee. Both observers were blinded to all clinical data and to each other’s measurements. For continuous variables, the mean of the two measurements was used for analysis, whereas for categorical variables, any disagreement was resolved through discussion. If consensus could not be achieved, the final decision was made in consultation with the senior author.

### 2.3. Statistical Analysis

All statistical analyses were performed with SAS version 9.4 (SAS Institute Inc., Cary, NC, USA). In initial between-group comparisons, normality of continuous variables was assessed using the Shapiro–Wilk test. Variables satisfying normality assumptions were compared using Student’s *t*-test, whereas the Mann–Whitney U test was applied for non-normally distributed variables. Categorical variables were compared using Pearson’s chi-square test, or Fisher’s exact test when more than 20% of cells had an expected frequency of ≤5. Because treatment allocation (isolated ACL reconstruction vs. combined ACL + ALL reconstruction) was determined historically rather than randomized, we addressed confounding by surgical indication using an inverse probability of treatment weighting (IPTW) based on a propensity score [44]. The propensity model included factors related to the surgical indication for ALL reconstruction—age, activity level, sex, and rotational knee laxity—and additionally incorporated preoperative radiographic osteoarthritis grade as a prognostic covariate. IPTW was then applied to create a weighted pseudo-population in which these baseline covariates were balanced across groups [44]. Covariate balance before and after IPTW was assessed using standardized mean differences, with values < 0.2 considered acceptable and indicative of minimal residual imbalance [45]. After IPTW adjustment, between-group comparisons were conducted using weighted Student’s *t*-tests for continuous variables and the Rao–Scott chi-square test for categorical variables, both of which appropriately account for the weighting scheme and the resulting pseudo-population created by IPTW. Interobserver reliability for radiographic measurements of continuous variables was evaluated using intraclass correlation coefficients (ICC) with 95% confidence intervals. Statistical significance was defined as *p* < 0.05.

## 3. Results

A total of 59 patients were included in this study (Group 1: 39 isolated ACL reconstructions; Group 2: 20 combined ACL and ALL reconstructions), and between-group comparisons were performed for variables included in the propensity model for IPTW. In the initial comparison, a statistically significant difference was observed in the preoperative pivot-shift test (*p* = 0.008); however, no significant difference was noted after IPTW, indicating that the overall balance of covariates was improved following weighting (Table 1).

Regarding clinical outcomes, no significant between-group differences were observed in any functional scores at the preoperative, 2-year postoperative, or final follow-up assessments. However, the incidence of surgical failure, as defined in this study, was significantly lower in Group 2 than in Group 1, and this difference was consistently observed both before and after IPTW adjustment (*p* = 0.044 for the initial comparison; *p* = 0.049 after IPTW application) (Table 2). For knee laxity, no significant between-group differences were observed either before or after IPTW application, except for the preoperative pivot-shift test in the initial comparison (*p* = 0.008) (Table 3).

Comparisons of radiologic parameters and intraoperative data were also performed. No significant between-group differences were observed in preoperative radiologic parameters or intraoperative findings. Among the postoperative assessments, most parameters showed no significant differences between groups; however, SNQ of the ACL graft evaluated by MRI at 1 year postoperatively was significantly lower in Group 2, and this finding was consistent both before and after IPTW adjustment (*p* = 0.046 for the initial comparison; *p* = 0.038 after IPTW application) (Table 4). The interobserver ICCs for the continuous radiological parameters ranged from 0.845 to 0.918.

## 4. Discussion

The principal finding of this study was that, in ACL reconstruction performed using allografts, the addition of ALL reconstruction resulted in more favorable clinical outcomes compared with ACL reconstruction alone. Patients who underwent the combined procedure demonstrated a lower incidence of surgical failure and improved graft maturity during the follow-up period. These findings suggest that, for patients for whom ACL reconstruction with allografts is planned, combined ALL reconstruction is recommended when performing ACL reconstruction in appropriately indicated cases.

The advantages of combined ACL and ALL reconstruction have been well established. The addition of ALL reconstruction to ACL reconstruction provides superior biomechanical stability of the knee joint compared with isolated ACL reconstruction and has been shown to yield distinct clinical benefits [1,2,3,4,5,6,46]. However, most previous studies have focused on cases in which autografts were used for ACL reconstruction, and limited information is available regarding procedures performed with allografts [1,2,3,4,5]. It has been theoretically recognized that autografts and allografts differ in several aspects, including the ligamentization process—which encompasses graft incorporation and revascularization—as well as in their mechanical properties [11,12,15]. Although a recent large cohort study has suggested that the graft source is not directly associated with clinical outcomes after ACL reconstruction [47], the potential disadvantages of allografts warrant careful consideration when interpreting postoperative results. Given the increasing adoption of ALL reconstruction in clinical practice [7], further investigation is warranted to clarify how combined ALL reconstruction influences the outcomes of ACL reconstruction using allografts, thereby prompting the design of the present study.

In this study, when allografts were used as the graft source for ACL reconstruction, the incidence of surgical failure was significantly lower in patients who underwent combined ACL and ALL reconstruction than in those who underwent isolated ACL reconstruction. Although no significant differences were observed when each parameter—functional scores and postoperative knee laxity—was analyzed separately, the reduced rate of surgical failure in the combined reconstruction group may indicate a clinically meaningful advantage of adding ALL reconstruction. These findings are consistent with previous studies using autografts for ACL reconstruction [1,2,3,4,5], despite variations in the definition of surgical failure across studies. Furthermore, although the difference did not reach statistical significance, patients who underwent combined reconstruction tended to exhibit less residual anteroposterior laxity postoperatively. Overall, these results suggest that the addition of ALL reconstruction to ACL reconstruction may confer potential clinical benefits, even when allografts are used as the graft source.

Beyond the clinical outcomes, this study demonstrated a difference in ACL graft maturity between the two groups. Patients who underwent additional ALL reconstruction exhibited significantly lower SNQ values of the ACL graft compared with those who received isolated ACL reconstruction, indicating a relatively greater degree of graft maturation. The SNQ of the ACL graft measured on MRI has been reported to correlate with the graft’s mechanical properties, showing a negative correlation with load to failure, tensile strength, and stiffness [48]. Accordingly, patients who underwent the combined procedure exhibited more advanced graft maturation and healing. This finding may be explained by the stress-sharing effect of the reconstructed ALL, which could reduce mechanical stress on the ACL graft during the healing period, thereby preventing microdamage or overstretching and promoting graft maturation. Beyond this mechanical explanation, the addition of ALL reconstruction may also have influenced biological healing responses from surrounding soft tissues, such as vascular ingrowth and cellular migration described in epiligament-related healing mechanisms, thereby contributing to the observed differences in graft maturation [16,49]. Although such biological processes were not directly evaluated in the present study, interpretation of our findings within the framework of the epiligament theory may provide a complementary biological context [16,49]. The difference in graft maturity observed between the two groups may, in turn, have contributed to the disparity in the incidence of surgical failure in this study. Although previous studies investigating the effect of anterolateral augmentation procedures, including ALL reconstruction, on ACL graft healing have reported conflicting results [50,51,52], it should be noted that the present study differed from most prior reports in that allografts were used as the graft source for ACL reconstruction, whereas autografts were predominantly used in previous investigations. Taken together, these findings suggest that combined ACL and ALL reconstruction may yield more favorable outcomes than isolated ACL reconstruction, not only in subjective but also in objective assessments.

As previously discussed, the clinical advantages of adding ALL reconstruction to ACL reconstruction have been well established. However, the present study holds particular significance as it is, to our knowledge, the first to analyze this combined procedure specifically in the context of allograft-based ACL reconstruction. By employing matched comparisons, this study provides a balanced comparison that minimizes confounding related to surgical indications and baseline characteristics. These findings may serve as meaningful clinical evidence supporting the potential benefits of performing combined ACL and ALL reconstruction when allografts are used as the graft source.

This study has several limitations. First, as this was a retrospective study, the potential risks of selection and information bias could not be fully eliminated. Second, the number of patients included in this study was relatively small, which may have introduced limited statistical power and increased susceptibility to sampling-related bias, including variability in data distribution for certain outcomes. However, because the analysis was limited to patients who underwent ACL reconstruction using allografts at a single institution, this limitation was inevitable. Furthermore, the statistical power of the outcome comparisons was dependent on the IPTW rather than the absolute sample size, given that the analysis was conducted using an IPTW-based pseudo-population. Third, the patients were grouped historically according to whether an additional ALL reconstruction was performed, and this grouping inherently reflected differences in the original surgical indications. Although the potential confounding effect of surgical indication was minimized by performing a matched analysis that controlled for related covariates, the possibility of residual confounding cannot be entirely excluded. Fourth, changes in clinical practice over the study period may have introduced unmeasured sources of variability. While the single-surgeon, single-center design ensured technical consistency, it may limit the generalizability of the findings and introduce selection bias. Finally, the SNQ, which was identified as a clinically relevant variable in this study, was measured only once at 1 year postoperatively. Considering that the ligamentization process of allografts may extend beyond 1 year, serial MRI evaluations would be required for a more precise assessment of graft maturity.

## 5. Conclusions

In ACL reconstruction using allografts, the addition of ALL reconstruction resulted in more favorable clinical and radiologic outcomes—particularly a lower incidence of surgical failure and greater graft maturity at 1 year postoperatively—compared with isolated ACL reconstruction.

## Figures and Tables

**Table 1:** Comparison of covariates used for IPTW.

Variable ^b^	Overall Cohort				After IPTW ^a^			
	Group 1 (N = 39)	Group 2 (N = 20)	*p* Value	SMD	Group 1 (N = 42.9)	Group 2 (N = 28.3)	*p* Value	SMD
Covariates used for IPTW								
Age, years	32.7 ± 16.1	31.5 ± 11.9	0.943	0.084	33.3 ± 16.7	33.3 ± 10.2	>0.999	<0.001
Pre-injury Tegner activity score	5.9 ± 1.8	6.3 ± 1.6	0.667	0.208	6.0 ± 1.7	6.0 ± 1.5	0.961	0.014
Sex ^c^			0.493	0.193			0.685	0.14
Male	28	16			31.0	18.6		
Female	11	4			11.9	9.7		
Preoperative Pivot-shift, grade ^c^			0.008				0.913	
1	5	1		0.277	4.5	4.2		0.133
2	25	6		0.727	22.9	13.6		0.104
3	9	13		0.932	15.5	10.5		0.016
Preoperative KL grade ^c^			0.347				0.174	
0	33	18		0.162	37.9	26.5		0.185
1	4	1		0.199	3.5	1.3		0.146
2	2	0		0.329	1.5	0		0.167
3	0	1		0.324	0	0.5		0.189

IPTW, inverse probability of treatment weighting; KL, Kellgren–Lawrence; SMD, standardized mean difference. ^a^ Adjusted for the following covariates: age, pre-injury Tegner activity score, sex, preoperative pivot-shift test, and preoperative Kellgren–Lawrence grade. ^b^ Values are presented as mean ± standard deviation unless otherwise indicated. ^c^ Values are presented as the number of patients.

**Table 2:** Comparison of demographic data and clinical outcomes.

Variable ^b^	Overall Cohort			After IPTW ^a^		
	Group 1 (N = 39)	Group 2 (N = 20)	*p* Value	Group 1 (N = 42.9)	Group 2 (N = 28.3)	*p* Value
Demographic data						
Age, years	32.7 ± 16.1	31.5 ± 11.9	0.943	33.3 ± 16.7	33.3 ± 10.2	>0.999
Pre-injury Tegner activity score	5.9 ± 1.8	6.3 ± 1.6	0.667	6.0 ± 1.7	6.0 ± 1.5	0.961
Sex			0.493			0.685
Male/Female	28/11	16/4		31.0/11.9	18.6/9.7	
Affected side ^c^			0.787			0.536
Right/Left	20/19	11/9		23.2/19.7	12.6/15.7	
Body Mass Index, kg/m^2^	23.8 ± 3.0	25.0 ± 3.0	0.088	23.7 ± 2.9	24.8 ± 2.8	0.213
Postoperative MRI ^c^			0.156			- ^e^
Yes/No	34/5	20/0		35.3/7.7	28.3/0	
Follow-up duration, year	4.1 ± 1.6	3.5 ± 1.4	0.179	4.1 ± 1.6	3.2 ± 1.4	0.06
Preoperative						
IKDC subjective score	54.2 ± 16.9	49.7 ± 19.2	0.359	53.9 ± 16.9	46.1 ± 16.8	0.091
Lysholm score	62.4 ± 25.1	68.4 ± 12.2	0.553	60.7 ± 23.9	67.7 ± 13.8	0.226
Tegner activity score	1.4 ± 1.0	1.8 ± 1.2	0.266	1.4 ± 1.0	1.7 ± 1.0	0.204
Postoperative 2-years						
IKDC subjective score	77.2 ± 14.5	74.4 ± 13.5	0.374	77.4 ± 14.5	73.7 ± 14.1	0.399
Lysholm score	87.9 ± 11.6	82.0 ± 14.0	0.125	87.5 ± 11.1	82.4 ± 15.3	0.267
Tegner activity score	3.7 ± 2.0	3.9 ± 1.5	0.702	3.7 ± 2.1	3.7 ± 1.4	0.994
Final follow-up						
IKDC subjective score	76.3 ± 16.3	75.9 ± 14.7	0.854	74.4 ± 17.5	73.1 ±14.5	0.783
Lysholm score	85.7 ± 12.8	85.7 ± 12.8	0.891	84.1 ± 13.5	82.5 ± 13.6	0.698
Tegner activity score	3.7 ± 1.9	3.8 ± 1.4	0.714	3.6 ± 1.8	3.6 ± 1.3	0.996
Surgical failure ^c,d^			0.044			0.049
Yes/No	11/28	1/19		14.0/28.9	2.0/26.3	
Revision surgery ^c,d^			>0.999			0.767
Yes/No	3/36	1/19		4.2/38.8	2.0/26.3	

IPTW, inverse probability of treatment weighting; MRI, magnetic resonance imaging; IKDC, International Knee Documentation Committee. ^a^ Adjusted for the following covariates: age, pre-injury Tegner activity score, sex, preoperative pivot-shift test, and preoperative Kellgren–Lawrence grade. ^b^ Values are presented as mean ± standard deviation unless otherwise indicated. ^c^ Values are presented as the number of patients. ^d^ Defined as cases identified during the follow-up period. ^e^ *p* value could not be estimated due to a computational limitation caused by zero frequency in one category.

**Table 3:** Comparisons of knee laxity.

Variable ^b^	Overall Cohort			After IPTW ^a^		
	Group 1 (N = 39)	Group 2 (N = 20)	*p* Value	Group 1 (N = 42.9)	Group 2 (N = 28.3)	*p* Value
Preoperative						
SSD in ATT, MM	8.1 ± 2.1	6.7 ± 3.3	0.409	8.0 ± 2.0	7.0 ± 2.7	0.111
Lachman test, grade ^c^			0.325			0.435
1/2/3	0/37/2	0/17/3		0/41.3/1.7	0/26.0/2.3	
Pivot-shift test, grade ^c^			0.008			0.913
1/2/3	5/25/9	1/6/13		4.5/22.9/15.5	4.2/13.6/10.5	
Postoperative 2-years						
SSD in ATT, MM	2.2 ± 2.7	2.1 ± 2.1	0.972	2.3 ± 2.7	2.2 ± 1.9	0.796
Lachman test, grade ^c^			0.8			0.557
0/1/2	24/13/2	14/5/1		26.1/15.2/1.6	20.1/6.2/2.0	
Pivot-shift test, grade ^c^			0.713			0.313
0/1/2/3	27/10/1/1	16/3/1/0		28.5/11.0/1.6/1.9	23.0/3.3/2.0/0	
Final follow-up						
SSD in ATT, MM	2.9 ± 2.7	1.9 ± 1.7	0.109	3.3 ± 2.9	1.9 ± 1.8	0.055
Lachman test, grade ^c^			0.237			0.102
0/1/2	18/15/6	14/5/1		17.2/18.1/7.7	20.5/5.9/2.0	
Pivot-shift test, grade ^c^			0.529			0.293
0/1/2	21/14/4	14/5/1		21.4/15.8/5.8	20.7/5.6/2.0	

IPTW, inverse probability of treatment weighting; SSD, side-to-side difference; ATT, anterior tibial translation; MM, manual maximum. ^a^ Adjusted for the following covariates: age, pre-injury Tegner activity score, sex, preoperative pivot-shift test, and preoperative Kellgren–Lawrence grade. ^b^ Values are presented as mean ± standard deviation unless otherwise indicated. ^c^ Values are presented as the number of patients.

**Table 4:** Comparison of radiological parameters and intraoperative data.

Variable ^b^	Overall Cohort			After IPTW ^a^		
	Group 1 (N = 39)	Group 2 (N = 20)	*p* Value	Group 1 (N = 42.9)	Group 2 (N = 28.3)	*p* Value
Preoperative						
KL grade ^c^			0.347			0.174
0/1/2/3	33/4/2/0	18/1/0/1		37.9/3.6/1.5/0	26.5/1.3/0/0	
Hip–Knee–Ankle angle, °	0.1 ± 3.7	0.7 ± 2.1	0.467	0.06 ± 3.6	0.7 ± 2.2	0.399
Posterior Tibial Slope, °	7.8 ± 2.3	8.0 ± 3.0	0.785	7.7 ± 2.1	8.6 ± 3.1	0.249
Lateral femoral condyle ratio, %	66.6 ± 3.0	66.5 ± 3.0	0.933	66.8 ± 2.9	66.2 ± 2.9	0.534
Intraoperative data						
Graft diameter, mm	8.7 ± 0.6	9.0 ± 0.5	0.123	8.7 ± 0.6	8.9 ± 0.6	0.392
Medial meniscus ^c^			0.892			0.826
Functional/Non-functional/Repair	26/2/11	12/1/7		28.3/1.8/12.9	18.1/0.5/9.7	
Lateral meniscus ^c^			0.106			0.351
Functional/Non-functional/Repair	33/0/6	13/0/7		36.8/0/6.2	21.3/0/7.0	
Tibiofemoral joint cartilage ^c^			0.999			0.545
Intact or low-grade lesion/High-grade lesion/Repair	36/1/2	18/1/1		39.3/0.8/2.8	27.0/0.8/0.5	
Postoperative 1-year						
ACL Graft maturity, SNQ ^d^	28.1 ± 62.2	5.1 ± 4.4	0.046	28.1 ± 61.2	4.6 ± 4.3	0.038
Postoperative 2-years						
KL grade ^c^			0.38			0.324
0/1/2/3	29/8/2/0	15/2/2/1		33.2/8.2/1.5/0	21.6/3.0/3.2/0.5	
Hip–Knee–Ankle angle, °	0.0 ± 3.4	−0.1 ± 2.4	0.816	0.4 ± 3.4	0.3 ± 2.8	0.924
Final follow-up						
KL grade ^c^			0.454			0.449
0/1/2/3	24/13/2/0	10/7/2/1		26.9/14.6/1.5/0	14.6/10.0/3.2/0.5	
Hip–Knee–Ankle angle, °	0.0 ± 3.4	−0.3 ± 2.5	0.762	0.5 ± 3.4	0.4 ± 3.2	0.978

IPTW, inverse probability of treatment weighting; KL, Kellgren–Lawrence; ACL, anterior cruciate ligament; SNQ, signal-to-noise quotient. ^a^ Adjusted for the following covariates: age, pre-injury Tegner activity score, sex, preoperative pivot-shift test, and preoperative Kellgren–Lawrence grade. ^b^ Values are presented as mean ± standard deviation unless otherwise indicated. ^c^ Values are presented as the number of patients. ^d^ Data available for patients who underwent 1-year postoperative MRI (34 in Group 1 and 20 in Group 1).

## Data Availability

The datasets analyzed during the current study are available upon reasonable request.
